# Association between the geriatric nutritional risk index and the risk of stroke in elderly patients with hypertension: A longitudinal and cohort study

**DOI:** 10.3389/fnut.2022.1048206

**Published:** 2022-12-06

**Authors:** Xintian Cai, Junli Hu, Wen Wen, Mengru Wang, Qing Zhu, Shasha Liu, Wenbo Yang, Yujie Dang, Jing Hong, Nanfang Li

**Affiliations:** Hypertension Center, Xinjiang Hypertension Institute, NHC Key Laboratory of Hypertension Clinical Research, Key Laboratory of Xinjiang Uygur Autonomous Region, Xinjiang Clinical Medical Research Center for Hypertension Diseases, People's Hospital of Xinjiang Uygur Autonomous Region, Urumqi, China

**Keywords:** geriatric nutritional risk index, stroke, elderly, hypertension, longitudinal cohort

## Abstract

**Objective:**

We aimed to investigate the association between the GNRI and the risk of stroke in elderly patients with hypertension.

**Methods:**

A total of 5312 elderly hypertensive patients free of history of stroke were included. Multivariate Cox models were used to calculate hazard ratios (HRs) and their 95% confidence intervals (CIs) for stroke and its subtypes.

**Results:**

The average time of follow-up was 3.8 years, and the median time was 3.2 years. We identified 640 individuals with stroke, of whom 526 had an ischemic stroke (IS) and 114 had a hemorrhagic stroke (HS). After adjusting for confounding variables, compared with participants in the lowest quartile of the GNRI, those in the third and fourth quartiles were associated with a decreased risk of stroke (adjusted HR 0.72, 95% CI 0.58–0.90, and adjusted HR 0.58, 95% CI 0.46–0.74, respectively, P for trend < 0.001). Similar results were found for IS and HS. Moreover, there were L-shaped associations of GNRI with new-onset HS (P for non-linearity = 0.034). Multiple sensitivity analyses and stratified analyses did not materially change the results.

**Conclusions:**

In summary, we found that a lower GNRI was associated with a higher risk of incident stroke in elderly hypertensive patients. Additional prospective data collection is required to confirm our findings.

## Introduction

Stroke, including ischemic stroke (IS) and hemorrhagic stroke (HS), is the leading cause of the global burden of disease (1). China has the highest burden of strokes in the world (2). There are still 250 million new instances of stroke each year in China, and that number is rising, even though the incidence and frequency of stroke have decreased globally (3). Hypertension has now been identified as the primary variable risk factor for stroke (4). Several large epidemiological surveys in China have shown that more than 50% of people over 60 years of age have hypertension (5). Therefore, identifying the residual risk of stroke and early risk stratification in elderly patients with hypertension is essential to more effectively tailoring risk reduction strategies.

Malnutrition is associated with a poor clinical prognosis in patients with various diseases (6). According to studies, malnutrition is significantly linked to increased levels of inflammatory response, arterial calcification, and atherosclerosis progression, which raises the possibility that it plays a key role in the emergence of cardiovascular disease (7, 8). The geriatric nutritional risk index (GNRI) is a simple, well-established nutrition assessment tool that uses serum albumin and body mass index (BMI) (9). Recent studies have shown that GNRI is associated with the development of atherosclerosis and an increased risk of cardiovascular mortality in older patients (10, 11). However, studies on GNRI as a predictor of new-onset stroke are still limited. Until now, only one cohort study has reported lower GNRI in hemodialysis patients as an independent risk factor for cerebral infarction and hemorrhage, and it is unclear whether this effect can be extended to older patients with hypertension (10). Therefore, GNRI may have important clinical implications for stroke risk stratification in hypertensive patients. In addition, the status of the dose-response relationship between GNRI and the risk of stroke and its subtypes in elderly hypertensive patients is uncertain.

Therefore, the present study is based on a cohort study aiming to investigate the association between GNRI and the risk of stroke and its subtypes in elderly hypertensive patients and to characterize the nature of the dose-response relationship.

## Materials and methods

### Study population

**Figure 1:**
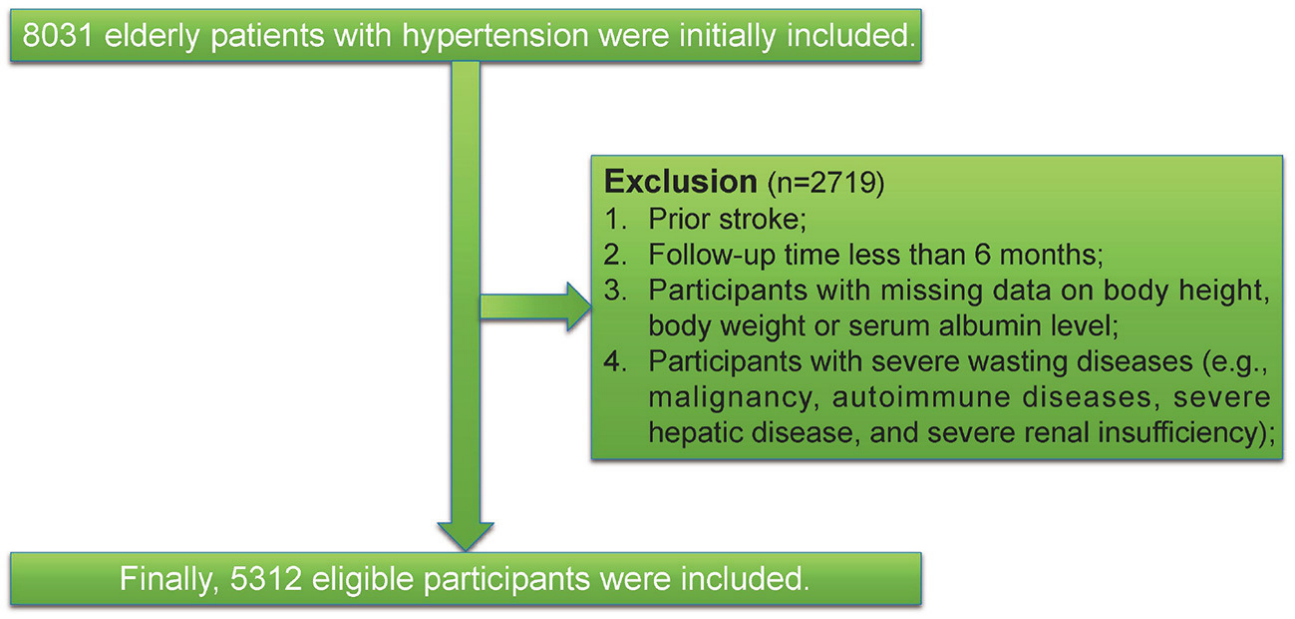
Study flowchart.

We conducted a cohort study at the People's Hospital of Xinjiang Uygur Autonomous Region, Xinjiang, China. All patients were either older than 60 years of age and were recruited between January 1, 2010, and December 31, 2021. First, we excluded patients who had < 6 months of follow-up or had the outcome at baseline. Second, we further excluded individuals with missing data on body height, body weight, or serum albumin level. Third, we excluded participants with severe wasting diseases (e.g., malignancy, autoimmune diseases, severe hepatic disease, and severe renal insufficiency). Finally, this left a final study population of 5,312 patients. Participant flow is shown in Figure 1. A comparison of baseline characteristics for in- and excluded participants are presented in Supplementary Table S1. This study was approved by the ethics committee of the People's Hospital of Xinjiang Uygur Autonomous Region (No. KY2021031901). A waiver of informed consent was granted due to the retrospective data collection. The study was reported as per the STROBE statement for observational cohort studies (12).

### Covariate collection and definitions

Data were abstracted electronically from the patient's medical records, including demographic characteristics, diagnoses according to the International Classification of Diseases 10th Revision (ICD-10), prescribed medications, and laboratory reports. Weight, height, heart rate, and blood pressure (BP) were measured using standard protocols. The BMI (kg/m^2^) was computed from the measured weight and height. Smoking status included categories of current smokers and non-smokers. Participants are classified as current drinkers and non-smokers. Blood samples were drawn after an overnight fast. The participants' prior medical histories were evaluated using ICD-10 codes. To ensure the accuracy of diagnoses, coronary heart disease (CHD) (I24 and I25), diabetes (E10-E14), atrial fibrillation (I48), and dyslipidemia (E78) were regarded as present if a participant was treated ≥ 2 times. To quantify the burden of comorbidities, the Charlson Comorbidity Index (CCI) was calculated as described previously (13). Prescription claims within the last year before the baseline defined concomitant medications. The list of concomitant medications included in the study is shown in Supplementary Table S2. The GNRI formula used was as follows: GNRI = (1.489 × albumin, g/l) + (41.7 × present/ideal body weight). Ideal weight was calculated using the Lorenz formulas: For males: height - 100 - [(height - 150)/4]. For females: height - 100 - [(height - 150)/2.5] (14).

### Follow-up and outcome measures

The primary outcome was the first occurrence of stroke (ischemic or hemorrhagic), either nonfatal or fatal. Secondary outcomes included the first ischemic stroke and the first hemorrhagic stroke. Methods of determination of incident stroke are described in the Supplementary material. Outcomes of events since participants enrolled in the study were determined through medical records, patient and family interview, contact with local disease and death registries, or access to the database of basic medical insurance. These data sources are linked using an individual national identification number assigned to each Chinese person for life. This number is replaced by a series number when provided for personal data analysis to anonymize the individual participant's data. Patients were followed from the date of enrollment to the end of the observation period, defined as the date of the last follow-up visit, the date of the first appearance of any study outcome, the date of death, or the end of the study period (December 31, 2021).

### Statistical analysis

Details of the missing covariates are shown in Supplementary Table S3. Missing values of covariates (all covariates were missing in < 6%) were imputed using multiple imputations by chained equations. Characteristics of study participants were expressed by GNRI quartiles. For differences in cumulative incidence between groups, we used Kaplan-Meier curves and the log-rank test. The multicollinearity test suggested that the variance inflation factors of all variables were less than five, confirming that the regression model was not affected by multicollinearity. The hazard ratio (HR) estimates and 95% confidence intervals (CI) were determined by the Cox regression models. Tests for non-linear associations were performed using restricted cubic spline regressions. We also performed subgroup analyses stratified by potential confounders. Sensitivity analyses were undertaken to evaluate the robustness of the results. First, to minimize the chance of reverse causation, we excluded events that occurred within 1 or 3 years after the baseline visit. Second, sensitivity analysis determined whether event risks remained stable after accounting for competing risks. Third, participants with CCI ≥2 were excluded to reduce confounding factors caused by associated comorbidity. Fourth, participants with atrial fibrillation were excluded. Lastly, to evaluate potential unmeasured confounding, we calculated E-values. Further analysis details are provided in the Supplementary material. Statistical analyses were performed using R software, version 4.1.1. Two-sided *P*-values < 0.05 were considered statistically significant.

## Results

### Baseline characteristics

**Figure 2:**
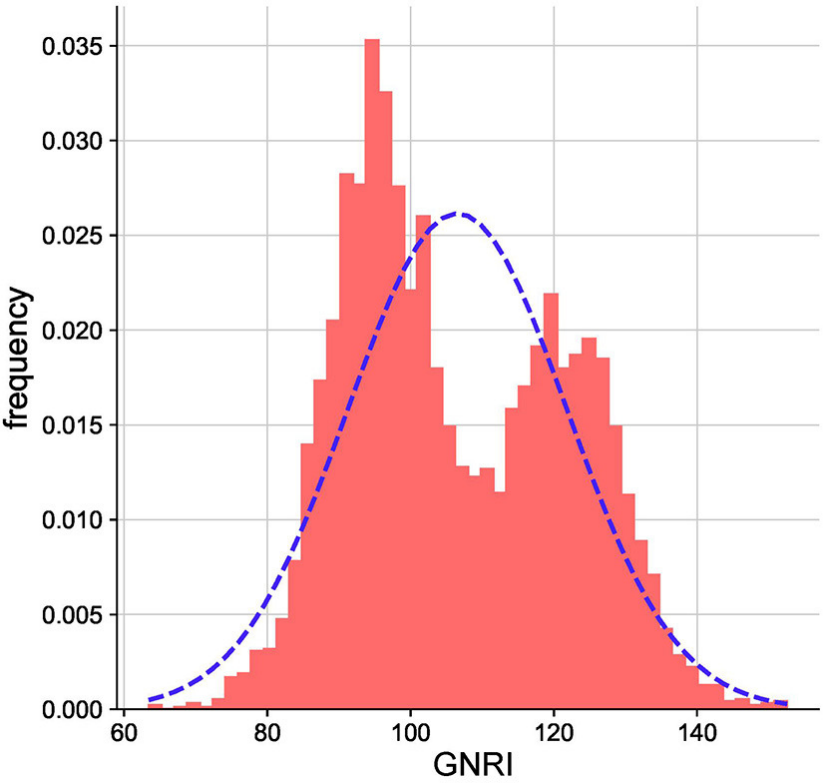
Distribution of GNRI among participants.

As illustrated in the flow chart (Figure 1), a total of 5312 participants were included in the current study. The average age of the study population was 66.5 years (SD 4.8). The GNRI was approximately normally distributed (Figure 2). Baseline characteristics of the study participants by GNRI quartiles are shown in Table 1. Participants with a lower GNRI tended to be women, have a higher duration of hypertension, a higher prevalence of dyslipidemia, take more statins and aspirin, have higher HbA1c, TC, TG, and LDL-C levels, and have lower HDL-C compared with participants in the quartile 4 group.

**Table 1:** Baseline characteristics stratified across quartiles of GNRI.

**Characteristics**	**GNRI quartiles**				**P-value**
	**Quartile 1**	**Quartile 2**	**Quartile 3**	**Quartile 4**	
	**( ≤ 94.33)**	**(94.34–103.17)**	**(103.18–119.61)**	**(≥119.63)**	
No. of participants	1327	1329	1328	1328	
Age, years	66.17 ± 4.70	66.67 ± 4.87	66.69 ± 4.82	66.43 ± 4.78	0.316
Male, *n* (%)	624 (47.02%)	732 (55.08%)	692 (52.11%)	696 (52.41%)	< 0.001
Current smoker, *n* (%)	396 (29.84%)	423 (31.83%)	381 (28.69%)	385 (28.99%)	0.283
Current drinker, *n* (%)	339 (25.55%)	381 (28.67%)	329 (24.77%)	353 (26.58%)	0.119
**Duration of hypertension, years**					< 0.001
< 5	789 (59.46%)	1065 (80.14%)	972 (73.19%)	1044 (78.61%)	
5–9	167 (12.58%)	71 (5.34%)	185 (13.93%)	183 (13.78%)	
≥10	371 (27.96%)	193 (14.52%)	171 (12.88%)	101 (7.61%)	
Heart rate, bpm	80.37 ± 9.73	80.51 ± 10.07	80.37 ± 9.80	80.50 ± 9.73	0.970
SBP, mmHg	144.12 ± 20.62	143.28 ± 20.49	143.73 ± 20.52	143.92 ± 19.54	0.738
DBP, mmHg	89.02 ± 14.52	88.68 ± 13.92	88.89 ± 14.18	88.91 ± 14.21	0.940
BMI, kg/m^2^	24.31 ± 3.30	24.41 ± 3.34	24.35 ± 3.32	24.19 ± 3.34	0.355
**Comorbid conditions**, ***n*** **(%)**					
Dyslipidemia	858 (64.66%)	807 (60.72%)	778 (58.58%)	787 (59.26%)	< 0.001
Atrial fibrillation	26 (1.96%)	27 (2.03%)	33 (2.48%)	40 (3.01%)	0.255
Coronary heart disease	215 (16.20%)	233 (17.53%)	218 (16.42%)	225 (16.94%)	0.799
Diabetes	358 (26.98%)	371 (27.92%)	385 (28.99%)	362 (27.26%)	0.662
**Charlson comorbidity index**					0.139
0	617 (46.50%)	579 (43.57%)	631 (47.52%)	594 (44.73%)	
1	391 (29.46%)	380 (28.59%)	347 (26.13%)	387 (29.14%)	
≥2	319 (24.04%)	370 (27.84%)	350 (26.36%)	347 (26.13%)	
**Laboratory tests**					
ALT, U/L	24.17 (15.00–35.59)	24.42 (15.75–35.07)	24.41 (15.93–35.63)	24.62 (14.50–35.90)	0.685
AST, U/L	21.00 (16.00–28.27)	21.40 (16.00–27.71)	21.21 (16.00–28.45)	21.37 (16.00–28.17)	0.929
GGT, U/L	27.79 (17.04–41.51)	28.00 (17.89–40.40)	29.05 (18.08–42.49)	28.68 (17.45–41.48)	0.230
Cr, μmol/L	69.92 ± 22.48	69.08 ± 21.63	70.05 ± 22.25	69.62 ± 22.86	0.686
UA, μmol/L	334.05 ± 91.20	334.06 ± 90.17	333.41 ± 91.31	331.81 ± 90.97	0.910
BUN, mmol/L	5.26 ± 1.51	5.28 ± 1.51	5.28 ± 1.51	5.33 ± 1.54	0.712
TC, mmol/L	4.61 ± 0.96	4.58 ± 0.96	4.51 ± 0.96	4.47 ± 0.99	0.005
TG, mmol/L	1.69 (1.12–2.48)	1.60 (1.08–2.32)	1.64 (1.15–2.49)	1.56 (1.08–2.32)	0.016
HDL-C, mmol/L	1.04 ± 0.27	1.07 ± 0.26	1.15 ± 0.26	1.19 ± 0.27	0.006
LDL-C, mmol/L	2.88 ± 0.81	2.77 ± 0.80	2.73 ± 0.80	2.70 ± 0.83	0.009
HbA1c, %	6.21 ± 1.08	6.20 ± 1.06	6.13 ± 1.05	6.10 ± 1.03	0.014
FPG, mmol/L	5.20 ± 1.35	5.23 ± 1.37	5.27 ± 1.48	5.22 ± 1.42	0.621
Hcy, μmol/L	15.11 ± 6.36	15.15 ± 6.23	15.24 ± 6.28	15.12 ± 6.41	0.949
**Concomitant medications**, ***n*** **(%)**					
Statins	652 (49.13%)	626 (47.10%)	585 (44.05%)	559 (42.09%)	< 0.001
Aspirin	955 (71.97%)	930 (69.98%)	903 (68.00%)	858 (64.61%)	< 0.001
ACEI/ARB	928 (69.93%)	937 (70.50%)	963 (72.52%)	923 (69.50%)	0.333
Beta-blocker	461 (34.74%)	463 (34.84%)	490 (36.90%)	487 (36.67%)	0.509
Calcium channel blockers	1036 (78.07%)	1023 (76.98%)	1077 (81.10%)	1048 (78.92%)	0.064
Diuretics	296 (22.31%)	290 (21.82%)	331 (24.92%)	288 (21.69%)	0.156
Insulin	126 (9.50%)	134 (10.08%)	132 (9.94%)	125 (9.41%)	0.921
Oral antidiabetic agents	222 (16.73%)	229 (17.23%)	249 (18.75%)	235 (17.70%)	0.565

GNRI, metabolic score for insulin resistance; SBP, systolic blood pressure; DBP, diastolic blood pressure; BMI, body mass index; ALT, alanine aminotransferase; AST, aspartate aminotransferase; GGT, gamma-glutamyl transferase; TC, total cholesterol; TG, triglyceride; HDL-C, high-density lipoprotein cholesterol; LDL-C, low-density lipoprotein cholesterol; HbA1c, hemoglobin A1c; FPG, fasting plasma glucose; Hcy, homocysteine; UA, uric acid; Cr, blood creatinine; BUN, blood urea nitrogen.

### Association between GNRI and total stroke and its subtypes

**Figure 3:**
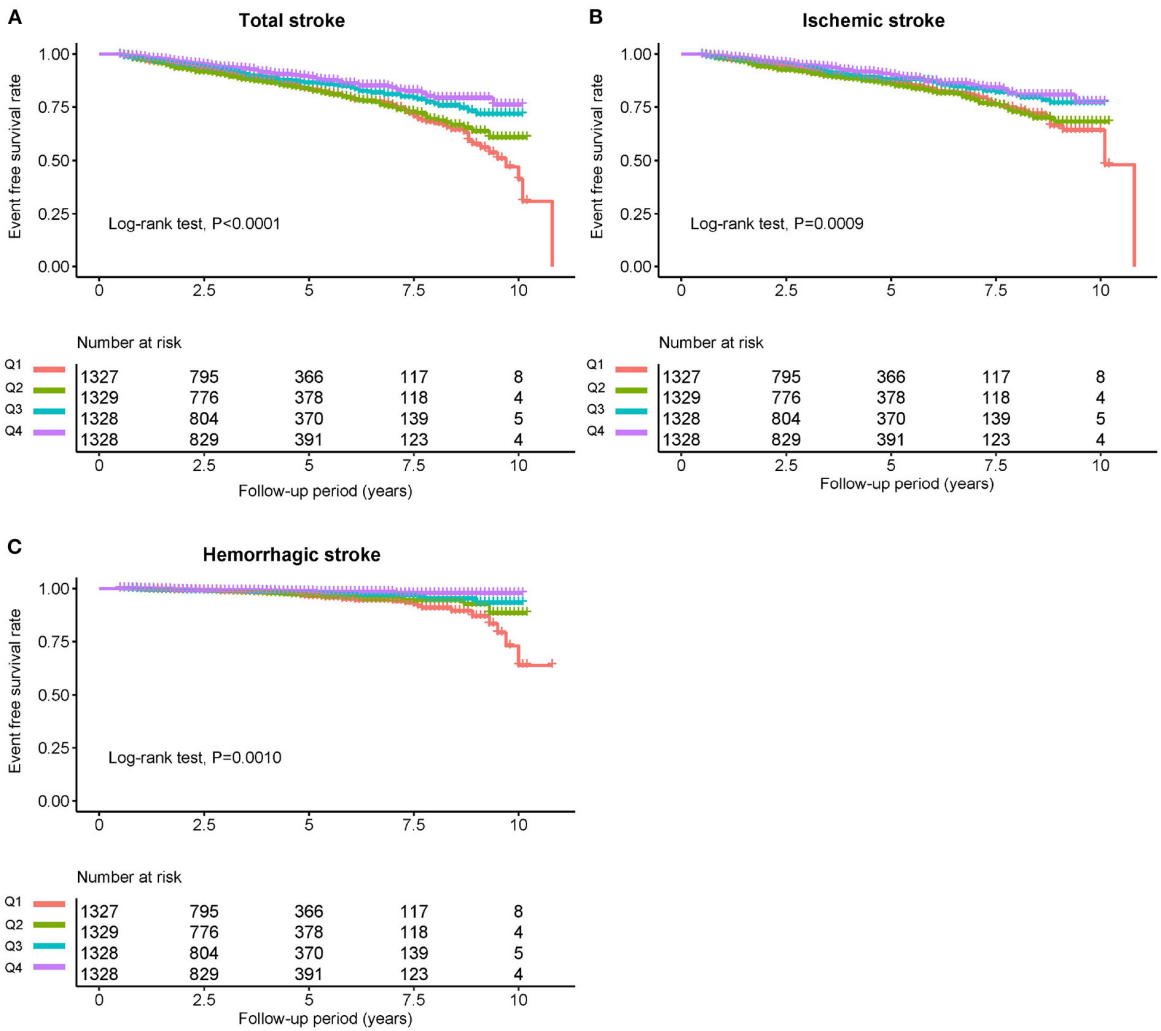
Kaplan-Meier survival curves for total stroke and individual outcomes based on GNRI quartiles. **(A)** Total stroke, **(B)** ischemic stroke, and **(C)** hemorrhagic stroke.

**Figure 4:**
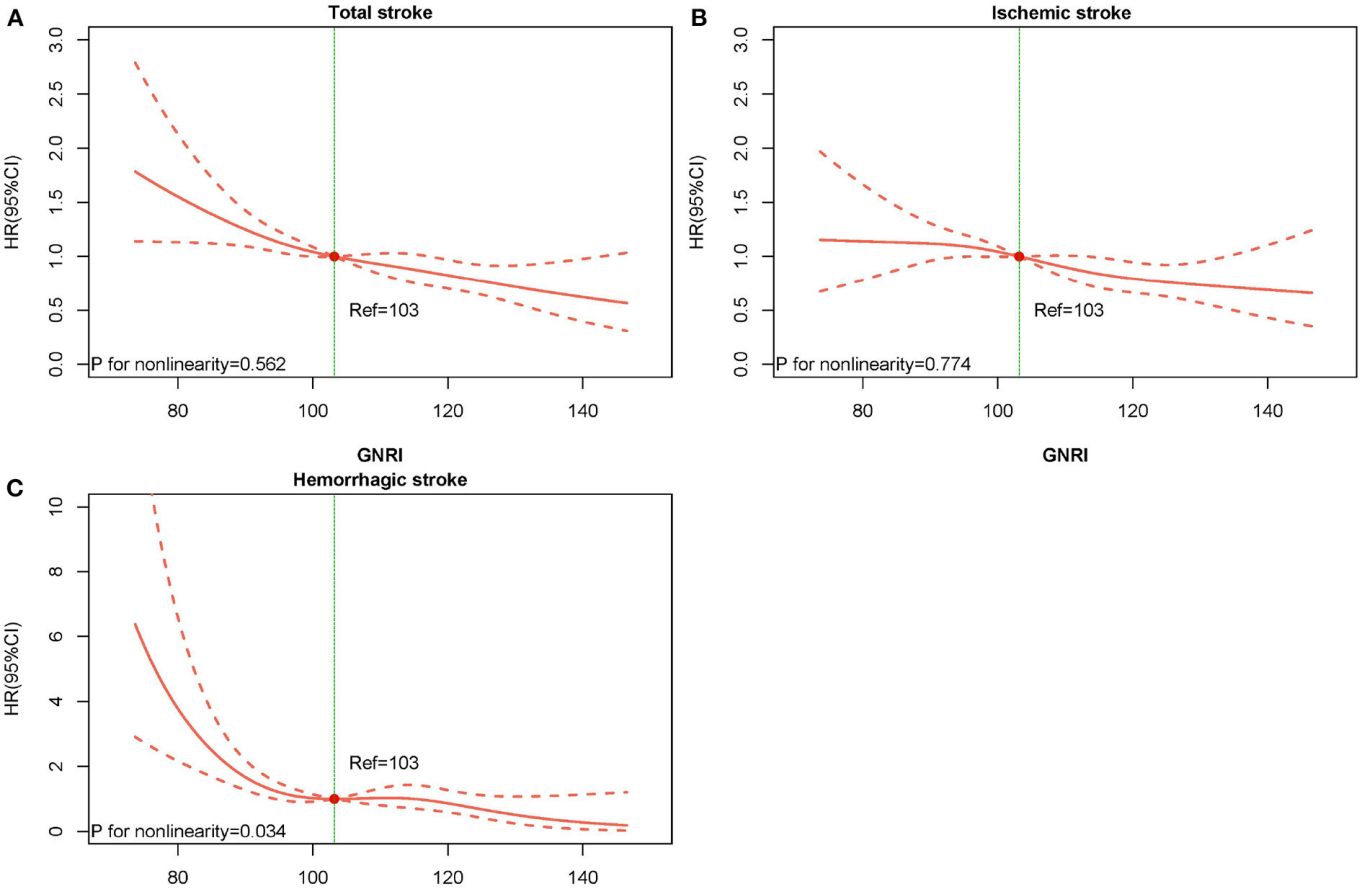
Dose-response association between GNRI and risk of stroke events. **(A)** Total stroke, **(B)** ischemic stroke, and **(C)** hemorrhagic stroke.

The average time of follow-up was 3.8 years, and the median time was 3.2 years. We identified 640 individuals with stroke, of which 526 had IS and 114 had HS. The Kaplan-Meier curve showed that participants in the quartile 1 group had a higher risk of total stroke, IS instead of HS than those in other groups (log-rank test, *P* < 0.001, Figure 3A; *P* = 0.001, Figure 3B; *P* = 0.001, Figure 3C). Overall, there was a significant inverse association of GNRI with the risk of first total stroke (Figure 4A) (per SD increment; full adjusted HR: 0.80; 95% CI: 0.73, 0.87). Consistently, when GNRI was assessed as quartiles, the full adjusted HRs of first stroke for participants in quartile 2, quartile 3, and quartile 4 were 0.94 (95% CI: 0.77, 1.15), 0.72 (95% CI: 0.58, 0.90), and 0.58 (95% CI: 0.46, 0.74) respectively, compared with those in quartile 1 (P for trend < 0.001) (Table 2). Similarly, a significant inverse association between GNRI and both IS and HS (Figure 4). Moreover, there were L-shaped associations of GNRI with new-onset HS (P for non-linearity = 0.034). Sensitivity analyses were conducted to verify the robustness of the reported findings. The results of sensitivity analyses were similar to those observed in the main analysis (Supplementary Tables S4–S9 and Supplementary Figure S1 in the Supplement).

**Table 2:** Association between GNRI and the incidence of outcomes.

**Exposure**	**Unadjusted**	**Model 1**	**Model 2**	**Model 3**
	**HR (95% CI)**	**HR (95% CI)**	**HR (95% CI)**	**HR (95% CI)**
**Total stroke**				
Per SD increment	0.81 (0.74, 0.87)	0.79 (0.73, 0.86)	0.80 (0.73, 0.87)	0.80 (0.73, 0.87)
**Quartiles**				
Q1	Ref	Ref	Ref	Ref
Q2	1.00 (0.82, 1.22)	0.95 (0.78, 1.16)	0.94 (0.77, 1.16)	0.94 (0.77, 1.15)
Q3	0.74 (0.60, 0.92)	0.71 (0.57, 0.88)	0.72 (0.58, 0.90)	0.72 (0.58, 0.90)
Q4	0.60 (0.48, 0.76)	0.58 (0.46, 0.73)	0.58 (0.46, 0.74)	0.58 (0.46, 0.74)
P for trend	< 0.001	< 0.001	< 0.001	< 0.001
**Ischemic stroke**				
Per SD increment	0.85 (0.78, 0.93)	0.84 (0.77, 0.92)	0.84 (0.77, 0.92)	0.84 (0.76, 0.91)
**Quartiles**				
Q1	Ref	Ref	Ref	Ref
Q2	1.07 (0.86, 1.34)	1.02 (0.81, 1.27)	1.00 (0.80, 1.26)	1.00 (0.80, 1.25)
Q3	0.79 (0.62, 1.00)	0.75 (0.59, 0.96)	0.76 (0.59, 0.96)	0.75 (0.59, 0.96)
Q4	0.69 (0.54, 0.88)	0.66 (0.51, 0.84)	0.65 (0.51, 0.84)	0.65 (0.50, 0.84)
P for trend	< 0.001	< 0.001	< 0.001	< 0.001
**Hemorrhagic stroke**				
Per SD increment	0.61 (0.50, 0.75)	0.61 (0.49, 0.75)	0.64 (0.52, 0.79)	0.64 (0.52, 0.79)
**Quartiles**				
Q1	Ref	Ref	Ref	Ref
Q2	0.76 (0.48, 1.20)	0.75 (0.47, 1.19)	0.78 (0.49, 1.24)	0.77 (0.48, 1.22)
Q3	0.54 (0.33, 0.89)	0.54 (0.33, 0.89)	0.58 (0.35, 0.96)	0.57 (0.35, 0.95)
Q4	0.34 (0.19, 0.61)	0.33 (0.19, 0.60)	0.38 (0.21, 0.69)	0.38 (0.21, 0.69)
P for trend	< 0.001	< 0.001	0.001	0.001

Model 1: adjusted for age, sex, BMI, heart rate, SBP, DBP, duration of hypertension, smoking, and drinking status.

Model 2: model 1 plus comorbid conditions.

Model 3: model 2 plus laboratory tests and concomitant medications.

SD, standard deviation; HR, hazard ratio; CI, confidence interval. Other abbreviations as presented in Table 1.

### Stratified analyses

**Figure 5:**
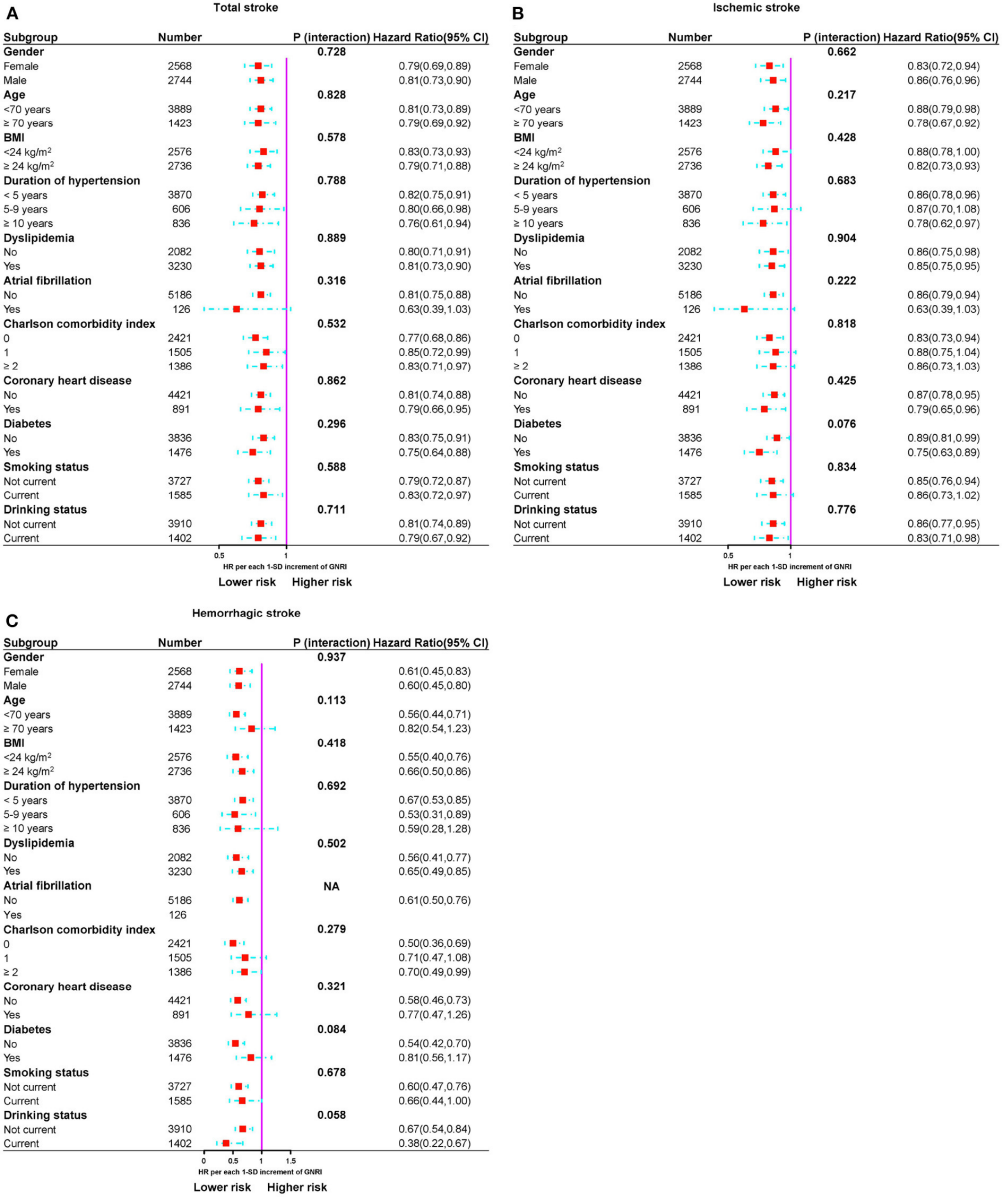
Subgroup analyses of the relationship between GNRI and risk of stroke events. **(A)** Total stroke, **(B)** ischemic stroke, and **(C)** hemorrhagic stroke.

Stratified analyses were performed to assess the association of GNRI (per SD increment) with total stroke and its subtypes, as provided in Figure 5. No interaction was found between subgroup variables and the association of GNRI with the risk of total stroke. Similar results were found for IS and HS.

### Incremental predictive value of GNRI

As illustrated in Table 3, according to C-statistic, risk prediction was improved by adding the GNRI to established risk factors (C-statistic increased from 0.613 to 0.648, *P* < 0.001). Moreover, according to continuous NRI and IDI, the GNRI significantly improved risk discrimination for total stroke [continuous NRI (95% CI): 0.118 (0.066–0.172), *P* < 0.001; IDI (95% CI): 0.017 (0.009–0.029), *P* < 0.001]. Furthermore, the DCA for the different models is shown in Supplementary Figure S2. The decision curves show that using a combination of GNRI features to predict total stroke increases the net benefit more than using established risk factors alone. Similar results were observed in IS and HS.

**Table 3:** Incremental predictive value of GNRI.

	**C-Statistic**	**P-value**	**cNRI**	**P-value**	**IDI**	**P-value**
	**Estimate (95% CI)**		**Estimate (95% CI)**		**Estimate (95% CI)**	
**Total stroke**						
Established risk factors	0.613 (0.590–0.636)		Reference		Reference	
Established risk factors + GNRI	0.648 (0.625–0.672)	< 0.001	0.118 (0.066–0.172)	< 0.001	0.017 (0.009–0.029)	< 0.001
**Ischemic stroke**						
Established risk factors	0.638 (0.612–0.663)		Reference		Reference	
Established risk factors + GNRI	0.658 (0.632–0.683)	0.005	0.096 (0.044–0.160)	< 0.001	0.012 (0.005–0.021)	0.033
**Hemorrhagic stroke**						
Established risk factors	0.673 (0.625–0.721)		Reference		Reference	
Established risk factors + GNRI	0.718 (0.670–0.766)	0.017	0.190 (0.057–0.311)	0.007	0.014 (0.005–0.052)	< 0.001

The established risk factorsl included age, sex, heart rate, duration of hypertension, SBP, DBP, smoking status, drinking status, comorbid conditions, and laboratory tests.

DI, integrated discrimination improvement; cNRI, continuous net reclassification improvement. Other abbreviations as presented in Table 1.

## Discussion

This study investigated the relationship between GNRI and incident stroke in elderly hypertensive patients. The findings revealed that the risk of stroke was significantly associated with baseline GNRI after adjusting for multiple confounders. In addition, a significant L-shaped dose-response relationship between GNRI and the risk of incident HS was observed, indicating a rapid increase in the risk of HS when GNRI was below 103. These findings were reliable in subgroup and multiple sensitivity analyses. Overall, the present study revealed that low CNRI was associated with a higher risk of incident stroke. To our knowledge, this is the first study to show an association between GNRI and the risk of incident stroke in a large retrospective cohort.

Aging is a condition that affects all people. One of the most susceptible demographics and one that is more likely to experience nutritional issues is the elderly (15). Similarly, malnutrition is an important independent risk factor for stroke, as is hypertension (16–19). In contrast to other clinical variables, nutritional status is a modifiable risk factor that physicians can act on. Therefore, appropriate tools are needed to assess the nutritional status of elderly patients with hypertension and to identify patients at risk to reduce their risk of stroke. Among multiple proxies of nutritional status, serum albumin levels and BMI are often used to evaluate nutritional status (20, 21). Hypoalbuminemia has been linked to acute and chronic inflammation, low BMI may be a sign of malnutrition, and both conditions may be a result of the loss of muscle and adipose tissue (22). In the general population, hypoalbuminemia and higher BMI have been reported as independent risk factors for stroke (23–26). Systemic edema, hepatic failure, and inflammation all have a negative influence on serum albumin levels. The status of body fluids also impacts body weight (27, 28). Consequently, assessing nutritional risk and prognosis solely based on albumin or BMI may not be sufficient. Simple hematological data (serum albumin) and anthropometric data can be used to compute the GNRI, a simplified form of the nutritional risk index (including height and weight) (14, 15). These indicators are readily available and can reduce information bias. Because of its objective nature, GNRI overcomes the problems of traditional nutrition indicators, including subjective issues such as mini-nutritional assessments (29). And GNRI correlates well with malnutrition-inflammation scores and has been regarded as one of the gold standards for nutritional assessment of elderly patients with chronic diseases (10, 30). There is evidence that patients with chronic illnesses, including chronic hemodialysis and peripheral vascular disease, have a lower GNRI (31–35). The results of Xiong et al. demonstrated that low GNRI levels were a strong predictor of cardiovascular and cerebrovascular events in patients with CKD (36). GNRI has also been reported to predict cardiovascular events, including cardiovascular disease mortality, in patients with heart failure (37). Furthermore, a study by Anzaki et al. (38) showed that low GNRI levels were associated with all-cause mortality and major adverse cardiovascular events after elective percutaneous coronary intervention (PCI). Cheng et al. (11) shown that in patients with chronic coronary artery occlusion (CTO) following PCI, the GNRI score at admission was a reliable predictor of adverse cardiovascular events. The prediction of cardiovascular events following PCI in patients with CTO was greatly enhanced by including the GNRI score into existing risk prediction algorithms (11). Elderly patients with hypertension are prone to multiple chronic diseases and may focus more on the primary disease, but nutritional support is mostly neglected. Our findings suggest that physicians may incorporate the identification of nutritional status into their daily practice. From prior research and the findings of the current study, we suggest that modestly increasing calorie and protein intake in malnourished elderly patients with hypertension may reduce the risk of stroke (39, 40). Further prospective intervention trials are needed to establish causality.

Although the underlying mechanisms remain unclear, there are some possible explanations. Oxidative stress and inflammation play key roles in the pathogenesis of stroke (41). First, serum albumin is a multifunctional protein that exerts neuroprotective effects in ischemic strokes, such as resisting antioxidants and reducing erythrocyte pressure levels (42, 43). According to Dziedzic et al. (43) stroke patients with decreased serum albumin levels had worse prognoses. Low albumin levels significantly enhanced the probability of recurrence in stroke patients and were related with poor outcome in all stroke subtypes (43, 44). Second, another potential mechanism may be attributed to the inflammatory response. According to a number of studies, inflammation frequently fosters a catabolic state that increases protein breakdown and slows protein synthesis, resulting in malnutrition and a decrease in GNRI (45). Additionally, it has been proven in the past that malnutrition is associated with higher than normal levels of inflammatory markers (46). Lower albumin levels are a result of the catabolic cytokines, muscle catabolism, and hunger suppression that are linked to chronic inflammatory disorders (47, 48). As a result, inflammation may be a key relationship between dietary status and the risk of cardiovascular disease (49). Severe malnutrition is closely associated with high levels of inflammation, and inflammation can increase the burden of atherosclerosis (50). At the same time, the inflammatory response reduces albumin synthesis, further inducing malnutrition (51). There may be a positive feedback loop between inflammation, malnutrition, immune defense, and adverse events, resulting in a vicious cycle. Therefore, the link between these three entities is also described as the malnutrition-inflammation-atherosclerosis syndrome (52, 53).

The strengths of this study lie in the novelty, the long observational period, and the well-characterized participants. Despite the aforementioned merits, several limitations of the present study merit discussion. First, it was observational and cannot establish causation. Second, the time-dependent changes of GNRI during the follow-up period were not assessed. Third, we didn't examine the predictive value of GNRI against more thorough nutrition evaluations. Despite adjustment for major confounding factors, the risk of residual unmeasured confounding remains possible. Finally, this study is limited to China and needs to be replicated in other different populations. Given the limitations inherent in this study, these results should be interpreted with caution but warrant further investigation in subsequent studies.

## Conclusion

In summary, we demonstrated that a lower GNRI was associated with a higher risk of incident stroke in elderly hypertensive patients. In addition, a significant L-shaped dose-response relationship between GNRI and the risk of incident HS was observed. Additional prospective data collection is required to confirm our findings.

## Data availability statement

The original contributions presented in the study are included in the article/Supplementary material, further inquiries can be directed to the corresponding author/s.

## Ethics statement

The studies involving human participants were reviewed and approved by People's Hospital of Xinjiang Uygur Autonomous Region. Written informed consent for participation was not required for this study in accordance with the national legislation and the institutional requirements.

## Author contributions

XC and JHu analyzed the data and wrote the manuscript. JHo, QZ, MW, YD, and WY helped with copyediting. XC and NL audited the data. SL, JHu, and XC conducted research. NL had primary responsibility for the final content of the manuscript. All authors read and approved the final manuscript.

## Funding

This research was supported by the Chinese Academy of Medical Sciences (2020-RW330-002).

## Conflict of interest

The authors declare that the research was conducted in the absence of any commercial or financial relationships that could be construed as a potential conflict of interest.

## Publisher's note

All claims expressed in this article are solely those of the authors and do not necessarily represent those of their affiliated organizations, or those of the publisher, the editors and the reviewers. Any product that may be evaluated in this article, or claim that may be made by its manufacturer, is not guaranteed or endorsed by the publisher.
