# Supplementary material for: Association between the geriatric nutritional risk index and the risk of stroke in elderly patients with hypertension: A longitudinal and cohort study
Source: Front Nutr. 2022 Dec 6;9:1048206. doi: 10.3389/fnut.2022.1048206 (PMC9763600; doi:10.3389/fnut.2022.1048206)
Supplement: Supplementary file 1 [file Data_Sheet_1.docx]

Supplementary Material

# Supplemental material and methods

**Confirmation of incident stroke cases**

Stroke was diagnosed following the WHO criteria as a constellation of neurologic deficits of sudden or rapid onset that lasted at least 24 hours or until death with no apparent cause other than that of vascular origin ^1^. Possible stroke cases were determined through medical records, patient and family interview, medical insurance documents, or death certificates. An expert panel of physicians reviewed medical records and adjudicated definite stroke cases according to clinical symptoms and confirmatory imaging findings. Stroke subtypes were carefully classified by physicians according to CT and/or MRI diagnoses into two major categories: a) ischemic stroke, including subtypes of large-artery occlusive infraction, lacunar infarction, cardioembolic infarction and other demonstrated cause of infraction according to the TOAST classification ^2^; b) hemorrhagic stroke, including subtypes of intracerebral hemorrhage and subarachnoid hemorrhage. Moreover, fatal stroke cases were identified by death certificates with International Classification of Diseases (ICD) codes (ICD-10 160, 161, 163, 164 and ICD-10 169.0, 169.1, 169.3, 169.4). The researchers crosschecked all the dates of stroke events through medical records and death certificates.

**Details of the statistical analyses**.

Details of the missing covariates are shown in Table S3. Missing values of covariates (all covariates were missing in <6%) were imputed using multiple imputations by chained equations. Participants were divided into four groups according to GNRI quartiles calculated at baseline. Differences in normally distributed continuous variables for basic characteristics across the four groups were analyzed using the variance, and non-normally distributed continuous variables by the Kruskal-Wallis test. Pearson's χ^2^ test was used for categorical variables.

For differences in cumulative incidence between groups, we used Kaplan-Meier curves and the log-rank test. We also tested whether there was severe multicollinearity among the variables in each test by calculating the variance inflation factor (VIF), which can potentially make effect size estimates unstable, reduce or eliminate statistical power, and cause the coefficients to switch signs. Multicollinearity analyses revealed that VIFs were smaller than 5 for all predictor variables, confirming that regression models were not affected by the presence of multicollinearity. Multivariable-adjusted Cox proportional hazards regression models were used to estimate the adjusted hazard ratios (aHR) and 95% confidence intervals (CI). In the first model, we adjusted for age, sex, BMI, heart rate, SBP, DBP, duration of hypertension, smoking, and drinking status. The second model was adjusted for model 1 plus dyslipidemia, atrial fibrillation, coronary heart disease, diabetes, and Charlson comorbidity index. The third model was adjusted for model 2 plus ALT, AST, GGT, Cr, UA, BUN, TC, TG, HDL-C, LDL-C, HbA1c, FPG, Hcy, use of statins, use of aspirins, use of insulin, use of oral antidiabetic drugs, and antihypertensive drugs. In addition, we also assessed the associations of GNRI with stroke subtypes, including IS and HS. In the trend test, the categorical variable was statistically examined as an ordinal variable (continuous variable) in the Cox regression model. We used restricted cubic splines to assess the potential nonlinear associations of GNRI with incident stroke and its subtypes, with 3 knots placed at the 5th, 50th, and 95th percentiles.

Moreover, we performed stratified analyses by baseline characteristics, including age (<70 or ≥70 years), sex (male or female), BMI (<24 or ≥24 kg/m^2^), current smokers (yes or no), current drinkers (yes or no), atrial fibrillation (yes or no), diabetes (yes or no), coronary heart disease (yes or no), CCI (0 or 1 or ≥2), duration of hypertension (<5 or 5-9 or ≥10 years), and hyperlipidemia (yes or no). Potential interactions were tested by adding multiplicative interaction terms into the models.

Sensitivity analyses were undertaken to evaluate the robustness of the results. First, to minimize the chance of reverse causation, we excluded events that occurred within 1 or 3 years after the baseline visit. Second, sensitivity analysis determined whether event risks remained stable after accounting for competing risks. Third, participants with CCI ≥2 were excluded to reduce confounding factors caused by associated comorbidity. Fourth, participants with atrial fibrillation were excluded. Lastly, to evaluate potential unmeasured confounding, we calculated E-values.

We further calculated the C-statistics, the continuous net reclassification improvement (NRI), and the integrated discrimination improvement (IDI) to assess the incremental value of GNRI for risk prediction beyond established risk factors. 95% CI for continuous NRI IDI and were estimated with 1000 bootstrap replications. Then, the benefits and improved performance of models with or without the GNRI were compared using decision curve analysis (DCA).

Statistical analyses were conducted using R, version 4.1.1 (R Foundation for Statistical Computing). Differences were considered significant when the two-sided P value was ≤ .05.

# Supplementary Figures and Tables

## 2.1 Supplementary Figures

**Figure S1.** **E-values for the observed associations between GNRI and clinical outcomes. A, total stroke; B, ischemic stroke; C, hemorrhagic stroke.**


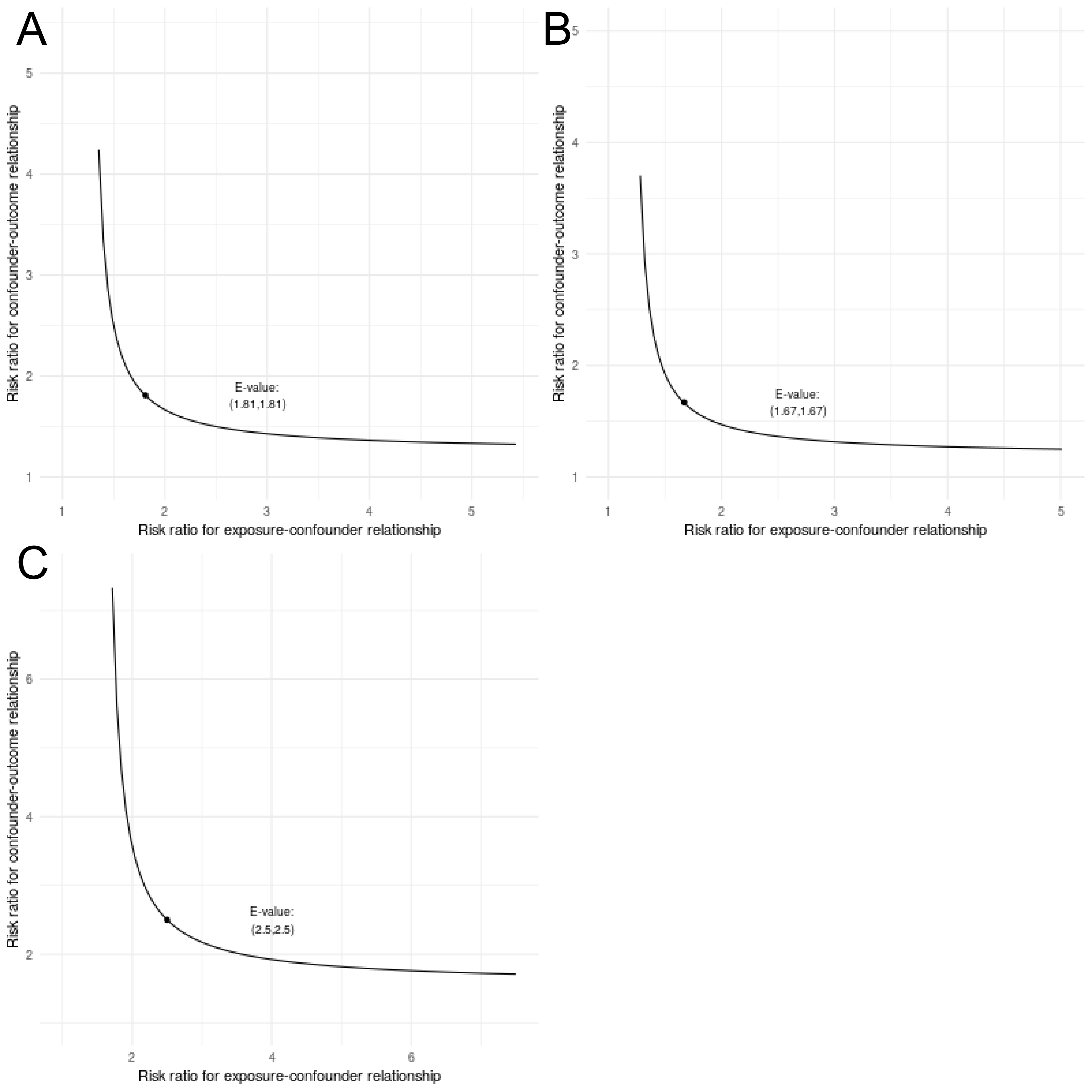


**Figure S2.** Decision curve analysis for established risk factors integrating GNRI features compared with established risk factors without using the GNRI. The red line representing the model integrating GNRI lies above the established risk factors. A, total stroke; B, ischemic stroke; C, hemorrhagic stroke.


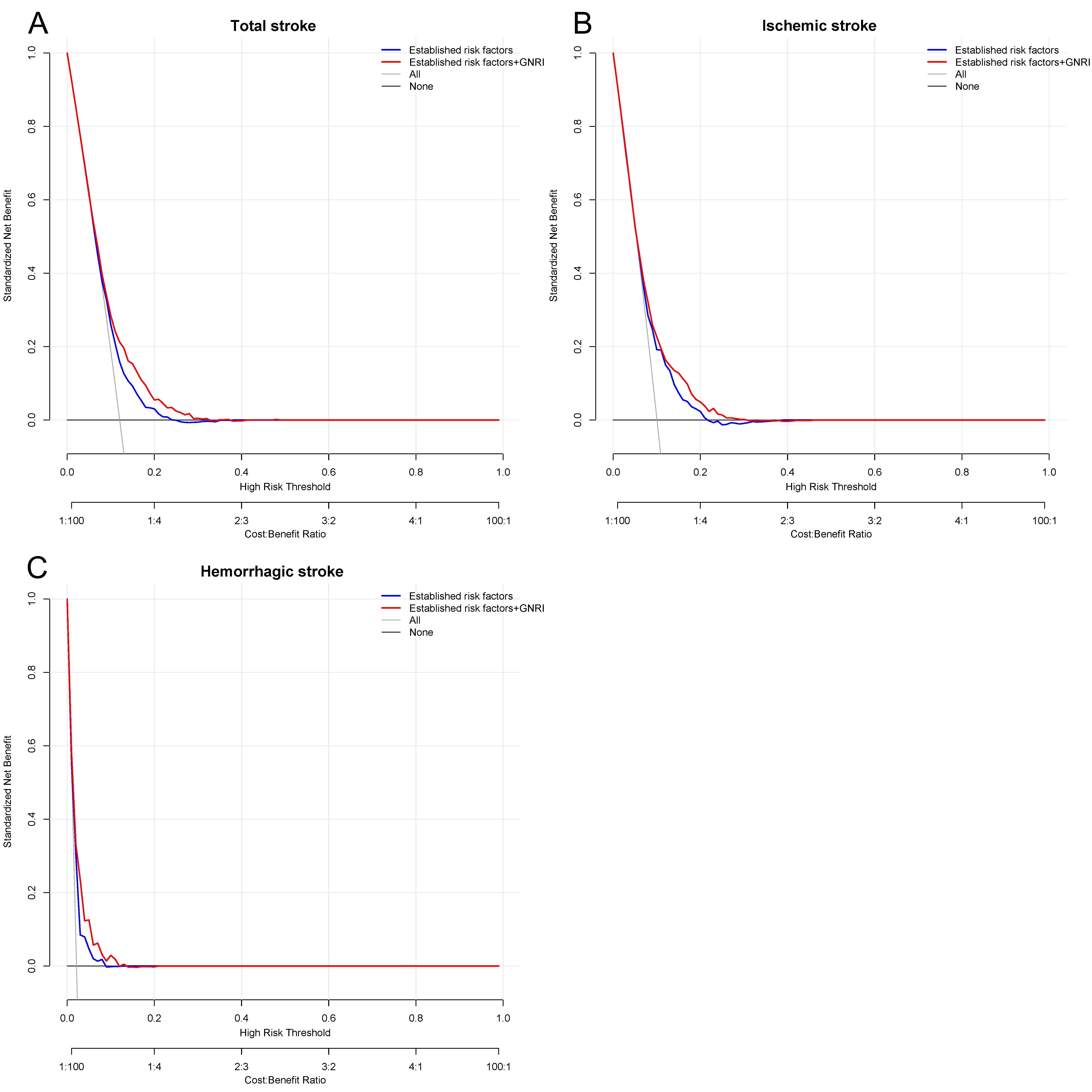


## 2.2 Supplementary Tables

**Table S1**. **Characteristics of the included and excluded population in the current study ***

| Characteristics | Included | Excluded |
| --- | --- | --- |
| No. of participants | 5312 | 2719 |
| Age, years | 66.49 ± 4.80 | 66.54 ± 4.83 |
| Male, n (%) | 2744 (51.66%) | 1438 (52.89%) |
| Current smoker, n (%) | 1585 (29.84%) | 815 (29.97%) |
| Current drinker, n (%) | 1402 (26.39%) | 713 (26.22%) |
| Duration of hypertension, years |  |  |
| < 5 | 3870 (72.85%) | 2075 (76.31%) |
| 5–9 | 606 (11.41%) | 289 (10.63%) |
| ≥ 10 | 836 (15.74%) | 355 (13.06%) |
| Heart rate, bpm | 80.44 ± 9.83 | 80.39 ± 9.84 |
| DBP, mmHg | 88.87 ± 14.21 | 88.72 ± 14.11 |
| SBP, mmHg | 143.76 ± 20.29 | 143.51 ± 20.48 |
| BMI, kg/m^2^ | 27.02 ± 3.69 | 27.06 ± 3.71 |
| Comorbid conditions, n (%) |  |  |
| Dyslipidemia | 3230 (60.81%) | 1699 (62.49%) |
| Atrial fibrillation | 126 (2.37%) | 56 (2.06%) |
| Coronary heart disease | 891 (16.77%) | 460 (16.92%) |
| Diabetes | 1476 (27.79%) | 769 (28.28%) |
| Charlson comorbidity index |  |  |
| 0 | 2421 (45.58%) | 1237 (45.49%) |
| 1 | 1505 (28.33%) | 748 (27.51%) |
| ≥ 2 | 1386 (26.09%) | 734 (27.00%) |
| Laboratory tests |  |  |
| ALT, U/L | 26.35 ± 14.82 | 26.42 ± 14.65 |
| AST, U/L | 22.51 ± 8.64 | 22.43 ± 8.68 |
| GGT, U/L | 30.82 ± 17.11 | 30.95 ± 17.17 |
| Cr, μmol/L | 69.67 ± 22.31 | 69.39 ± 21.73 |
| UA, μmol/L | 333.33 ± 90.89 | 333.87 ± 90.89 |
| BUN, mmol/L | 5.29 ± 1.52 | 5.29 ± 1.51 |
| TC, mmol/L | 4.50 ± 0.96 | 4.50 ± 0.96 |
| TG, mmol/L | 1.88 ± 1.12 | 1.89 ± 1.13 |
| HDL-C, mmol/L | 1.06 ± 0.26 | 1.06 ± 0.26 |
| LDL-C, mmol/L | 2.79 ± 0.81 | 2.78 ± 0.80 |
| HbA1c, % | 6.16 ± 1.05 | 6.19 ± 1.07 |
| FPG, mmol/L | 5.23 ± 1.41 | 5.24 ± 1.41 |
| Hcy, µmol/L | 15.16 ± 6.32 | 15.13 ± 6.27 |
| Concomitant medications, n (%) |  |  |
| Statins | 2422 (45.59%) | 1234 (45.38%) |
| Aspirin | 3646 (68.64%) | 1854 (68.19%) |
| Beta-blocker | 1901 (35.79%) | 959 (35.27%) |
| Calcium channel blockers | 4184 (78.77%) | 2139 (78.67%) |
| ACEI/ARB | 3751 (70.61%) | 1943 (71.46%) |
| Insulin | 517 (9.73%) | 272 (10.00%) |
| Oral antidiabetic agents | 935 (17.60%) | 483 (17.76%) |
| Diuretics | 1205 (22.68%) | 630 (23.17%) |

* Values are presented as mean ± standard deviation for continuous variables and n (%) for categorical variables.

Abbreviations as presented in Table 1.

**Table S2**. **List of medications included in the study.**

| Drug class | Drug name |
| --- | --- |
| Aspirin | Aspirin |
| Beta-blocker | Atenolol, bisoprolol, carvedilol, metoprolol, propranolol |
| Angiotensin-converting enzyme inhibitors or angiotensin receptor blockers | Azilsartan, candestartan, captopril, enalapril, fosinopril, irbesartan, losartan, olmesartan, ramipril telmisartan, valsartan |
| Calcium channel blockers | Amlodipine, diltiazem, felodipine, lercanidipine, nifedipine,  verapamil |
| Diuretics | Acetazolamide, amiloride, benzyl hydrochlorothiazide, bumetanide, furosemide, hydrochlorothiazide, indapamide, spironolactone |
| Statin | Atorvastatin, fluvastatin, pitavastatin, rosuvastatin, simvastatin |
| Oral antidiabetic agents | Metformin, glipizide, gliclazide, glimepiride, glyburide, alogliptin, linagliptin, sitagliptin, vidagliptin, saxagliptin, acarbose, nateglinide, meglitinide, repaglinde, pioglitzone,dulaglutide, exenatide, liraglutide |
| Insulin | Rapid, short, intermediate and long-acting insulins |

**Table S3**. **Counts and proportions of missing data.**

| Variables | Missing (%) |
| --- | --- |
| Age | 0 |
| Male | 0 |
| Current smoker | 0 |
| Current drinker | 0 |
| Duration of hypertension | 0 |
| Heart rate | 130 (2.45%) |
| SBP | 135 (2.54%) |
| DBP | 110 (2.07%) |
| BMI | 0 |
| Comorbid conditions |  |
| Dyslipidemia | 0 |
| Atrial fibrillation | 0 |
| Coronary heart disease | 0 |
| Diabetes | 0 |
| Charlson comorbidity index | 0 |
| Laboratory tests |  |
| ALT | 187 (3.52%) |
| AST | 177 (3.33%) |
| GGT | 162 (3.05%) |
| Cr | 239 (4.50%) |
| UA | 160 (3.01%) |
| BUN | 207 (3.90%) |
| TC | 171 (3.22%) |
| TG | 249 (4.69%) |
| HDL-C | 164 (3.09%) |
| LDL-C | 157 (2.96%) |
| HbA1c | 282 (5.31%) |
| FPG | 281 (5.29%) |
| Hcy | 192 (3.61%) |
| Concomitant medications |  |
| Statins | 0 |
| Aspirin | 0 |
| ACEI/ARB | 0 |
| Beta-blocker | 0 |
| Calcium channel blockers | 0 |
| Diuretics | 0 |
| Insulin | 0 |
| Oral antidiabetic agents | 0 |

Abbreviations as presented in Table 1.

**Table S4**. **Sensitivity analysis of excluding events occurred < 1 year of follow-up.**

| Exposure | Unadjusted | Model 1 | Model 2 | Model 3 |
| --- | --- | --- | --- | --- |
|  | HR (95% CI) | HR (95% CI) | HR (95% CI) | HR (95% CI) |
| **Total stroke** |  |  |  |  |
| Per SD increment | 0.81 (0.74, 0.88) | 0.79 (0.72, 0.87) | 0.80 (0.73, 0.88) | 0.80 (0.73, 0.88) |
| Quartiles |  |  |  |  |
| Q1 | Ref | Ref | Ref | Ref |
| Q2 | 0.98 (0.79, 1.23) | 0.93 (0.75, 1.17) | 0.94 (0.75, 1.17) | 0.93 (0.75, 1.17) |
| Q3 | 0.72 (0.56, 0.91) | 0.68 (0.54, 0.87) | 0.70 (0.55, 0.89) | 0.70 (0.55, 0.89) |
| Q4 | 0.62 (0.49, 0.80) | 0.59 (0.46, 0.76) | 0.62 (0.48, 0.80) | 0.61 (0.48, 0.79) |
| **Ischemic stroke** |  |  |  |  |
| Per SD increment | 0.86 (0.78, 0.94) | 0.84 (0.76, 0.93) | 0.85 (0.77, 0.94) | 0.84 (0.76, 0.93) |
| Quartiles |  |  |  |  |
| Q1 | Ref | Ref | Ref | Ref |
| Q2 | 1.07 (0.84, 1.37) | 1.01 (0.79, 1.29) | 1.01 (0.79, 1.29) | 1.00 (0.78, 1.28) |
| Q3 | 0.77 (0.59, 1.00) | 0.73 (0.56, 0.95) | 0.74 (0.56, 0.96) | 0.74 (0.56, 0.96) |
| Q4 | 0.72 (0.55, 0.94) | 0.68 (0.52, 0.90) | 0.70 (0.53, 0.92) | 0.69 (0.52, 0.91) |
| **Hemorrhagic stroke** |  |  |  |  |
| Per SD increment | 0.59 (0.47, 0.74) | 0.59 (0.47, 0.74) | 0.63 (0.50, 0.80) | 0.63 (0.50, 0.80) |
| Quartiles |  |  |  |  |
| Q1 | Ref | Ref | Ref | Ref |
| Q2 | 0.69 (0.42, 1.15) | 0.69 (0.41, 1.14) | 0.73 (0.44, 1.21) | 0.71 (0.43, 1.19) |
| Q3 | 0.49 (0.28, 0.86) | 0.49 (0.28, 0.86) | 0.55 (0.31, 0.96) | 0.54 (0.31, 0.94) |
| Q4 | 0.34 (0.18, 0.63) | 0.34 (0.18, 0.63) | 0.40 (0.21, 0.76) | 0.40 (0.21, 0.76) |

Model 1: adjusted for age, sex, BMI, heart rate, SBP, DBP, duration of hypertension, smoking, and drinking status.

Model 2: model 1 plus comorbid conditions.

Model 3: model 2 plus laboratory tests and concomitant medications.

**Abbreviations:** SD, standard deviation; HR, hazard ratio; CI, confidence interval. Other abbreviations as presented in Table 1.

**Table S5.** **Sensitivity analysis of excluding events occurred < 3 year of follow-up.**

| Exposure | Unadjusted | Model 1 | Model 2 | Model 3 |
| --- | --- | --- | --- | --- |
|  | HR (95% CI) | HR (95% CI) | HR (95% CI) | HR (95% CI) |
| **Total stroke** |  |  |  |  |
| Per SD increment | 0.75 (0.67, 0.85) | 0.74 (0.66, 0.84) | 0.74 (0.65, 0.84) | 0.74 (0.65, 0.84) |
| Quartiles |  |  |  |  |
| Q1 | Ref | Ref | Ref | Ref |
| Q2 | 0.87 (0.65, 1.17) | 0.83 (0.62, 1.11) | 0.83 (0.62, 1.11) | 0.83 (0.61, 1.11) |
| Q3 | 0.67 (0.50, 0.92) | 0.64 (0.47, 0.88) | 0.65 (0.47, 0.88) | 0.65 (0.48, 0.89) |
| Q4 | 0.50 (0.36, 0.70) | 0.47 (0.34, 0.67) | 0.48 (0.34, 0.67) | 0.47 (0.34, 0.67) |
| **Ischemic stroke** |  |  |  |  |
| Per SD increment | 0.83 (0.73, 0.95) | 0.81 (0.71, 0.93) | 0.80 (0.70, 0.92) | 0.80 (0.70, 0.92) |
| Quartiles |  |  |  |  |
| Q1 | Ref | Ref | Ref | Ref |
| Q2 | 0.97 (0.70, 1.35) | 0.91 (0.66, 1.27) | 0.90 (0.65, 1.26) | 0.91 (0.65, 1.27) |
| Q3 | 0.78 (0.55, 1.10) | 0.73 (0.51, 1.03) | 0.71 (0.50, 1.01) | 0.72 (0.51, 1.02) |
| Q4 | 0.64 (0.44, 0.92) | 0.60 (0.42, 0.86) | 0.58 (0.40, 0.85) | 0.58 (0.40, 0.84) |
| **Hemorrhagic stroke** |  |  |  |  |
| Per SD increment | 0.46 (0.34, 0.64) | 0.46 (0.34, 0.64) | 0.51 (0.37, 0.71) | 0.50 (0.37, 0.69) |
| Quartiles |  |  |  |  |
| Q1 | Ref | Ref | Ref | Ref |
| Q2 | 0.65 (0.35, 1.19) | 0.66 (0.36, 1.21) | 0.72 (0.39, 1.34) | 0.69 (0.37, 1.29) |
| Q3 | 0.38 (0.19, 0.77) | 0.39 (0.19, 0.79) | 0.45 (0.22, 0.91) | 0.41 (0.20, 0.85) |
| Q4 | 0.14 (0.05, 0.41) | 0.14 (0.05, 0.41) | 0.18 (0.06, 0.52) | 0.17 (0.06, 0.50) |

Model 1: adjusted for age, sex, BMI, heart rate, SBP, DBP, duration of hypertension, smoking, and drinking status.

Model 2: model 1 plus comorbid conditions.

Model 3: model 2 plus laboratory tests and concomitant medications.

**Abbreviations:** SD, standard deviation; HR, hazard ratio; CI, confidence interval. Other abbreviations as presented in Table 1.

**Table S6.** **Sensitivity analysis was conducted using the Fine-Gray competing risk model considering non-stroke deaths as competing risk events.**

| Exposure | Unadjusted | Model 1 | Model 2 | Model 3 |
| --- | --- | --- | --- | --- |
|  | SHR (95% CI) | SHR (95% CI) | SHR (95% CI) | SHR (95% CI) |
| **Total stroke** |  |  |  |  |
| Per SD increment | 0.81 (0.74, 0.87) | 0.80 (0.73, 0.86) | 0.80 (0.74, 0.87) | 0.80 (0.74, 0.87) |
| Quartiles |  |  |  |  |
| Q1 | Ref | Ref | Ref | Ref |
| Q2 | 1.00 (0.81, 1.22) | 0.95 (0.78, 1.17) | 0.95 (0.77, 1.16) | 0.94 (0.77, 1.16) |
| Q3 | 0.74 (0.60, 0.92) | 0.71 (0.57, 0.89) | 0.72 (0.58, 0.90) | 0.72 (0.58, 0.90) |
| Q4 | 0.60 (0.48, 0.76) | 0.58 (0.46, 0.73) | 0.59 (0.46, 0.74) | 0.59 (0.46, 0.74) |
| **Ischemic stroke** |  |  |  |  |
| Per SD increment | 0.85 (0.78, 0.93) | 0.84 (0.77, 0.92) | 0.84 (0.77, 0.92) | 0.84 (0.77, 0.92) |
| Quartiles |  |  |  |  |
| Q1 | Ref | Ref | Ref | Ref |
| Q2 | 1.07 (0.86, 1.34) | 1.02 (0.81, 1.28) | 1.01 (0.81, 1.26) | 1.00 (0.80, 1.26) |
| Q3 | 0.79 (0.62, 1.01) | 0.75 (0.59, 0.96) | 0.76 (0.59, 0.97) | 0.75 (0.59, 0.96) |
| Q4 | 0.69 (0.54, 0.88) | 0.66 (0.52, 0.85) | 0.66 (0.51, 0.85) | 0.65 (0.51, 0.84) |
| **Hemorrhagic stroke** |  |  |  |  |
| Per SD increment | 0.61 (0.50, 0.75) | 0.61 (0.49, 0.75) | 0.64 (0.52, 0.79) | 0.64 (0.52, 0.79) |
| Quartiles |  |  |  |  |
| Q1 | Ref | Ref | Ref | Ref |
| Q2 | 0.75 (0.48, 1.19) | 0.75 (0.47, 1.18) | 0.78 (0.49, 1.24) | 0.77 (0.48, 1.22) |
| Q3 | 0.54 (0.32, 0.88) | 0.54 (0.32, 0.88) | 0.58 (0.35, 0.96) | 0.57 (0.34, 0.95) |
| Q4 | 0.34 (0.19, 0.61) | 0.33 (0.19, 0.60) | 0.38 (0.21, 0.70) | 0.38 (0.21, 0.69) |

Model 1: adjusted for age, sex, BMI, heart rate, SBP, DBP, duration of hypertension, smoking, and drinking status.

Model 2: model 1 plus comorbid conditions.

Model 3: model 2 plus laboratory tests and concomitant medications.

**Abbreviations:** SD, standard deviation; SHR, subdistribution hazard ratio; CI, confidence interval. Other abbreviations as presented in Table 1.

**Table S7**. **Sensitivity analysis of excluding participants with CCI ≥2.**

| Exposure | Unadjusted | Model 1 | Model 2 | Model 3 |
| --- | --- | --- | --- | --- |
|  | HR (95% CI) | HR (95% CI) | HR (95% CI) | HR (95% CI) |
| **Total stroke** |  |  |  |  |
| Per SD increment | 0.79 (0.72, 0.87) | 0.78 (0.71, 0.86) | 0.78 (0.71, 0.86) | 0.80 (0.73, 0.87) |
| Quartiles |  |  |  |  |
| Q1 | Ref | Ref | Ref | Ref |
| Q2 | 0.98 (0.78, 1.24) | 0.93 (0.74, 1.18) | 0.92 (0.73, 1.16) | 0.94 (0.77, 1.15) |
| Q3 | 0.71 (0.55, 0.91) | 0.68 (0.53, 0.88) | 0.68 (0.53, 0.88) | 0.72 (0.58, 0.90) |
| Q4 | 0.59 (0.45, 0.77) | 0.56 (0.43, 0.73) | 0.56 (0.42, 0.73) | 0.58 (0.46, 0.74) |
| **Ischemic stroke** |  |  |  |  |
| Per SD increment | 0.85 (0.76, 0.94) | 0.83 (0.75, 0.92) | 0.83 (0.74, 0.92) | 0.84 (0.76, 0.91) |
| Quartiles |  |  |  |  |
| Q1 | Ref | Ref | Ref | Ref |
| Q2 | 1.13 (0.87, 1.47) | 1.06 (0.82, 1.38) | 1.05 (0.81, 1.36) | 1.00 (0.80, 1.25) |
| Q3 | 0.78 (0.59, 1.04) | 0.75 (0.56, 0.99) | 0.74 (0.56, 0.98) | 0.75 (0.59, 0.96) |
| Q4 | 0.70 (0.52, 0.93) | 0.66 (0.49, 0.88) | 0.64 (0.48, 0.87) | 0.65 (0.50, 0.84) |
| **Hemorrhagic stroke** |  |  |  |  |
| Per SD increment | 0.57 (0.44, 0.74) | 0.57 (0.44, 0.73) | 0.59 (0.45, 0.77) | 0.64 (0.52, 0.79) |
| Quartiles |  |  |  |  |
| Q1 | Ref | Ref | Ref | Ref |
| Q2 | 0.56 (0.31, 0.99) | 0.55 (0.31, 0.98) | 0.56 (0.31, 0.99) | 0.77 (0.48, 1.22) |
| Q3 | 0.46 (0.25, 0.84) | 0.46 (0.25, 0.83) | 0.49 (0.27, 0.90) | 0.58 (0.35, 0.95) |
| Q4 | 0.30 (0.15, 0.60) | 0.29 (0.15, 0.60) | 0.33 (0.16, 0.67) | 0.38 (0.21, 0.69) |

Model 1: adjusted for age, sex, BMI, heart rate, SBP, DBP, duration of hypertension, smoking, and drinking status.

Model 2: model 1 plus comorbid conditions.

Model 3: model 2 plus laboratory tests and concomitant medications.

**Abbreviations:** SD, standard deviation; HR, hazard ratio; CI, confidence interval. Other abbreviations as presented in Table 1.

**Table S8**. **Sensitivity analysis of excluding individuals with prevalent atrial fibrillation at baseline.**

| Exposure | Unadjusted | Model 1 | Model 2 | Model 3 |
| --- | --- | --- | --- | --- |
|  | HR (95% CI) | HR (95% CI) | HR (95% CI) | HR (95% CI) |
| **Total stroke** |  |  |  |  |
| Per SD increment | 0.81 (0.75, 0.88) | 0.80 (0.74, 0.87) | 0.80 (0.74, 0.87) | 0.80 (0.74, 0.87) |
| Quartiles |  |  |  |  |
| Q1 | Ref | Ref | Ref | Ref |
| Q2 | 1.00 (0.82, 1.23) | 0.95 (0.77, 1.17) | 0.95 (0.77, 1.16) | 0.94 (0.77, 1.16) |
| Q3 | 0.75 (0.61, 0.94) | 0.72 (0.58, 0.90) | 0.73 (0.59, 0.92) | 0.73 (0.59, 0.92) |
| Q4 | 0.60 (0.48, 0.76) | 0.58 (0.46, 0.73) | 0.58 (0.46, 0.74) | 0.58 (0.46, 0.74) |
| **Ischemic stroke** |  |  |  |  |
| Per SD increment | 0.86 (0.79, 0.94) | 0.85 (0.77, 0.93) | 0.85 (0.77, 0.93) | 0.84 (0.77, 0.93) |
| Quartiles |  |  |  |  |
| Q1 | Ref | Ref | Ref | Ref |
| Q2 | 1.08 (0.86, 1.36) | 1.02 (0.81, 1.28) | 1.01 (0.80, 1.27) | 1.00 (0.80, 1.26) |
| Q3 | 0.81 (0.63, 1.03) | 0.77 (0.60, 0.98) | 0.77 (0.60, 0.99) | 0.77 (0.60, 0.98) |
| Q4 | 0.69 (0.54, 0.90) | 0.66 (0.51, 0.86) | 0.66 (0.51, 0.85) | 0.66 (0.51, 0.85) |
| **Hemorrhagic stroke** |  |  |  |  |
| Per SD increment | 0.61 (0.50, 0.76) | 0.61 (0.50, 0.75) | 0.64 (0.52, 0.79) | 0.64 (0.52, 0.79) |
| Quartiles |  |  |  |  |
| Q1 | Ref | Ref | Ref | Ref |
| Q2 | 0.76 (0.48, 1.20) | 0.75 (0.47, 1.18) | 0.78 (0.49, 1.24) | 0.77 (0.48, 1.22) |
| Q3 | 0.55 (0.33, 0.90) | 0.55 (0.33, 0.90) | 0.58 (0.35, 0.96) | 0.57 (0.35, 0.95) |
| Q4 | 0.34 (0.19, 0.61) | 0.34 (0.19, 0.61) | 0.38 (0.21, 0.69) | 0.38 (0.21, 0.69) |

Model 1: adjusted for age, sex, BMI, heart rate, SBP, DBP, duration of hypertension, smoking, and drinking status.

Model 2: model 1 plus comorbid conditions (except atrial fibrillation).

Model 3: model 2 plus laboratory tests and concomitant medications.

**Abbreviations:** SD, standard deviation; HR, hazard ratio; CI, confidence interval. Other abbreviations as presented in Table 1.

**Table S9**. **E-values for the observed associations between GNRI and clinical outcomes.**

|  | Total stroke | Ischemic stroke | Hemorrhagic stroke |
| --- | --- | --- | --- |
| Observed association* (per SD increment) | 0.80 (0.73, 0.87) | 0.84 (0.76, 0.91) | 0.64 (0.52, 0.79) |
| E-value for point estimate | **1.81** | **1.67** | **2.50** |
| E-value for confdence interval | 1.56 | 1.43 | 1.85 |

*The observed associations are the fully adjusted hazard ratios (95% confidence intervals) shown in Table 2 and are presented here for reference.

**References**

1. Walker AE, Robins M, Weinfeld FD. The national survey of stroke. Clinical findings. Stroke. 1981;12:I13-44.

2. Adams HP Jr, Bendixen BH, Kappelle LJ, et al. Classification of subtype of acute ischemic stroke. Definitions for use in a multicenter clinical trial. TOAST. Trial of Org 10172 in Acute Stroke Treatment. Stroke. 1993;24(1):35-41.
